# Happy fifth birthday, *eClinicalMedicine*

**DOI:** 10.1016/j.eclinm.2023.102121

**Published:** 2023-07-24

**Authors:** eClinicalMedicine

July 2023, marks our 5-year anniversary and, looking back, a lot has happened since our inaugural issue went live in July 2018. Our team grew from two team members to now eight including, six Senior Editors, one Deputy Editor and one Editor-in-Chief. We received our first impact factor just 1 year ago and although this is just a partial measurement of our reputation, we are delighted to share that 1 year later we still have a strong Journal Impact Factor of 15.1. We are also very proud that our journal is now producing regular podcasts where we invite our authors to share their research and provide a platform to discuss the impact and relevance of their findings published with us. Our podcasts so far have reached listeners from 181 different countries with over 40k downloads.

One thing remains constant for our journal and that is our mission and dedication to help frontline health professionals navigate the complex and rapid health transitions facing societies worldwide. We aspire to be a catalyst to rethink and reframe the future of health and health care, with the ultimate goal of strengthening health systems as core institutions. We are only able to do this because of our authors and reviewers who contribute their hard work to the journal. Over the years, we have published several high-impact papers covering various clinical topics like public and global health, infectious diseases, psychiatry and oncology. Our published content comes from all over the world, including research from Asia, Africa, Europe, the Americas, and Oceania.

We are very proud of the work we have dedicated to our collections of research on an essential and underserved clinical need. Over the years, we have published three collections. Our first collection was published in March, 2020 and was titled Gender Equality and Health. The collection highlighted gender disparities and inequality across the world and to call for change of health systems, with a multipronged focus on women. Based on this research we launched an update of the collection in November, 2022, to renew the call for action for a gender equitable society especially in the aftermath of the COVID-19 pandemic, which exacerbated the value and treatment of women worldwide.

In June, 2021, we released our two-part Racial Inequity in Health collection which outlines and discusses racial and ethnic inequality across global settings. In this collection we curated research showing that health disparities are caused by structural racism and without tailored action the inequalities are maintained and reinforced. By highlighting these issues, we aimed to empower policy makers and health-care workers to prioritise health inequalities among minoritised ethnic groups at research, clinical, and governmental levels.

Our most recent collection entitled Reframing Obesity in Health Care and Ending Weight Stigma: Presenting Evidence for Change was published in April this year. The research presented in this collection investigated the prevalence and impact of weight bias in health care and challenged the preconceived notions about obesity. We also share evidence on how obesity should be diagnosed and further discuss the implications of defining obesity as a disease. We are especially proud to provide insights into the experiences of individuals living with the obesity which was also discussed in detail in one of our podcasts.

As part of our celebrations for this month we have commissioned commentaries linked to some of our most influential published work, asking the authors to look back at their papers and to outline the impact of the study on policies and guidelines, clinical practice, or health in general. We were able to review the impact of the 2017 International Council of Cardiovascular Prevention and Rehabilitation global audit on the cardiovascular rehabilitation programme availability globally and to assess the programme capacity and quality of services. Furthermore, we looked back on the capacity of maternity services in the state of Victoria, Australia, to implement, embed, and sustain a culturally specific caseload midwifery model of care. And finally, we reflect on the disproportional impact of the COVID-19 pandemic on people from minority ethnic backgrounds. We would like to invite you to read the commentaries in our July anniversary issue.

While we are very proud of our work in the past, we also want to give a brief overview of what to expect from *eClinicalMedicine* in the future. We are very excited to announce our future Series entitled Care of Preterm or Low Birthweight Infants which will include health policy pieces, as well as our joint Review Series with *The Lancet Global Health* entitled Maternal Health in the Perinatal Period and Beyond. Furthermore, we are currently working on a Review Series on Meaningful Endpoints in Oncology Trials. This Series aims to highlight the need for better surrogate endpoints that reflect real benefit. We were very excited to discuss this Series and related work in our newest podcast in which we interviewed Bishal Gyawali to celebrate the 5-year anniversary of the journal by looking back at his most prominent research and looking forward by highlighting important projects to come. Additionally, *eClinicalMedicine* will give a special emphasis on cancer in Asia with two cross-journal Series including *The Lancet Regional Health – Western Pacific* and *The Lancet Regional Health – Southeast Asia* or *The Lancet Child & Adolescent Health* focussing on lung or paediatric cancer respectively.

It has been 5 very exciting years for our journal. We would like to end our Editorial by giving credit to our authors and peer-reviewers, whose expertise and hard work enable us to give voice to essential, early evidence.

